# Smartphone-based retinal camera modified for ROP screening

**DOI:** 10.1186/s40942-025-00725-x

**Published:** 2025-10-14

**Authors:** Victor Ribeiro de Sant’Ana, Kellen Cristiane do Vale Lúcio, Alef José Fogaça, Nathalia Bertini Bonini, Eliane Chaves Jorge

**Affiliations:** 1 Botucatu Medical School, São Paulo State University (UNESP), Botucatu, São Paulo Brazil; 2 Department of Surgical Specialties and Anesthesiology (Ophthalmology Division), Botucatu Medical School, São Paulo State University (UNESP), Botucatu, São Paulo, Brazil

**Keywords:** Retinopathy of prematurity, Screening, Prevention of blindness, Telemedicine, Low-cost technology

## Abstract

**Background:**

Retinopathy of prematurity (ROP) is a leading cause of preventable childhood blindness worldwide, particularly affecting infants in resource-limited settings. Although indirect binocular ophthalmoscopy (IBO) is considered the definitive method for ROP screening, its broad adoption is restricted by the scarcity of skilled professionals and the practical difficulties involved in performing exams at the patient’s bedside. Smartphone-based retinal imaging is emerging as a viable, low-cost alternative for telemedicine-based ROP screening in underserved regions.

**Methods:**

This cross-sectional study was conducted from June 2022 to December 2023 in a Brazilian tertiary hospital. Preterm infants (GA ≤ 32 weeks and/or BW ≤ 1500 g) underwent standard ROP screening using IBO and a modified smartphone-based retinal camera (Phelcom Eyer^®^). Images of five retinal fields per eye were acquired by a single examiner, both with and without scleral indentation, which was performed when necessary to visualize the peripheral retina. A masked retina specialist graded image quality and classified ROP stages. Sensitivity, specificity, predictive values, and agreement with IBO (Cohen’s kappa) were calculated.

**Results:**

Seventy-one preterm infants (142 eyes) were included. ROP was diagnosed in 14 infants (19.7%), with six requiring treatment. Among the 337 images obtained, 69% were rated as excellent and 31% as acceptable; all were gradable. Compared to IBO, the device demonstrated a sensitivity of 82.0% and specificity of 100.0% without scleral indentation. When indentation was used—selectively, to improve visualization of Zone III—sensitivity increased to 91.3% and the negative predictive value to 98.3%. Agreement between methods was almost perfect (κ = 0.94). Only one transient episode of bradycardia occurred during indentation.

**Conclusions:**

The modified Phelcom Eyer^®^ camera demonstrated high diagnostic performance for ROP screening, particularly when complemented by selective scleral indentation to enhance peripheral retinal assessment. Its portability, affordability, and integration with telemedicine platforms make it a valuable tool for expanding ROP screening in resource-limited settings with restricted access to specialists.

## Introduction

Retinopathy of prematurity (ROP) is a condition of abnormal retinal vascular development affecting preterm infants arising from the interruption of physiological vasculogenesis due to premature birth [1–3]. It is a multifactorial disease and a leading cause of childhood blindness worldwide, particularly in low and middle-income countries. Early detection and treatment are crucial in preventing progression to severe, irreversible stages [4].

The global incidence of ROP-related blindness varies by country and is closely linked to socioeconomic conditions and the quality of neonatal care [5]. An increasing number of preterm infants at risk for ROP is observed, especially in developing regions such as Asia, the Middle East, and Latin America [6]. 

Fundoscopic examination in preterm infants requires technical skill. It may be time-consuming due to small ocular anatomy, respiratory instability, frequent bradycardia, use of invasive devices, and infection control precautions in cases of sepsis. Scleral indentation, often necessary for peripheral retinal assessment, also demands close monitoring of vital signs. Although considered the gold standard for ROP screening, indirect ophthalmoscopy has limitations, including a long learning curve, examiner dependence, lack of standardized documentation, and limited accessibility in resource-limited neonatal units [7–9]. 

The advent of portable pediatric retinal cameras has facilitated early diagnosis and remote evaluation of ROP through teleophthalmology since the early 2000s [10, 11]. However, the high cost of traditional systems such as RetCam limits their implementation in public health systems [11, 12]. 

Smartphone-based retinal imaging systems have emerged as alternatives due to their ease of use, portability, and reduced cost compared to traditional methods. Examples of such devices include the RetinaScope, MII RetCam, and various “Do It Yourself” (DIY) smartphone adapters used in studies for ROP screening [13–16]. This study aimed to assess the sensitivity and specificity of such a device—the Phelcom Eyer^®^—for ROP screening in preterm neonates.

## Methods

### Study design and setting

This was a cross-sectional, single-center study conducted from June 2022 to December 2023 at the Neonatal Intensive Care Unit (NICU), Intermediate Care Nursery, and Outpatient Ophthalmology Clinic of the Hospital das Clínicas, Botucatu Medical School – UNESP, Brazil.

### Ethical approval

The study was approved by the Research Ethics Committee of Botucatu Medical School - UNESP (protocol no. 5.625.864). Informed consent was obtained from the parents or legal guardians of all participants.

### Participants

Preterm infants admitted to the NICU were included if they met the following criteria: gestational age (GA) ≤ 32 weeks and/or birth weight (BW) ≤ 1500 g. Neonates were excluded if they had media opacities preventing fundus examination, congenital ocular anomalies that impaired ROP staging, or if informed consent was not provided. General information on the preterm infants, including sex, GA, BW, and ROP status, was documented.

### ROP screening

All enrolled neonates underwent standard ROP screening between the 4th and 6th weeks of life, by a trained ophthalmologist using indirect binocular ophthalmoscopy (IBO) and a 28-diopter lens after pupillary dilation with 0.5% tropicamide and 2.5% phenylephrine eye drops. The exams were repeated at intervals determined by the Brazilian ROP screening guidelines [17]. ROP was categorized according to the International Classification of Retinopathy of Prematurity [18, 19]. Clinical outcomes were defined as the onset of any stage of ROP (requiring no treatment) or severe ROP that required treatment. Each child was classified according to the most advanced ROP stage observed. The indications for treatment were based on the Early Treatment for Retinopathy of Prematurity study (ETROP) criteria [19]. 

### Imaging acquisition

**Fig. 1:**
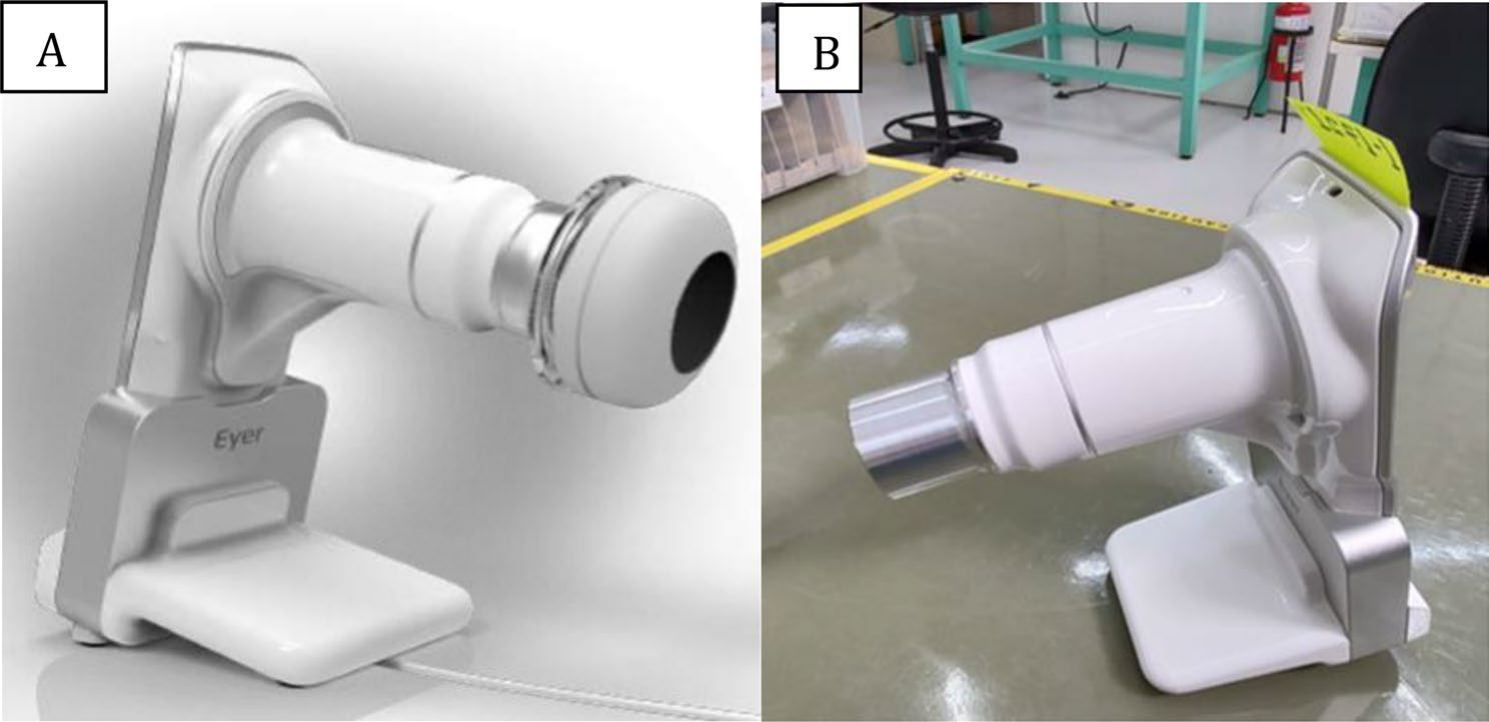
Phelcom Eyer^®^ smartphone-based portable camera. **A**: original version. **B**: modified version

Retinal images were captured using a modified version of the Phelcom Eyer^®^ smartphone-based portable camera (Samsung S9, 12 MP, Phelcom Technologies, São Carlos, SP, Brazil). The device was adapted for neonatal exams, with the removal of the original acrylic eye guard (Fig. 1). All images were acquired by the same examiner, who was masked to the IBO results. The imaging protocol included the examination of five retinal fields: posterior pole, temporal, nasal, superior, and inferior quadrants. Scleral indentation was applied, when necessary, to visualize the peripheral retina (Zone III). All examinations for each eye were performed on the same day, under topical anesthesia, and in a darkened room.

### Image evaluation and classification

Captured images were anonymized and graded by a masked retina specialist for all clinical and ophthalmoscopic findings related to the stage and zone of ROP, according to the International Classification of Retinopathy of Prematurity [18, 19]. The grader assessed the photograph quality by using the following criteria: excellent, acceptable (if it was overexposed, underexposed, or out of focus), and not gradable (out of focus or obscured by glare or motion artifacts).

### Learning curve analysis

The Image acquisition time by the examiner of both eyes was recorded for all preterm infants. Along the learning curve, the average acquisition time decreased from 25 to 10 min per neonate, indicating a need for training in the use of the smartphone-based retinal imaging systems.

### Outcomes and statistical analysis

Statistical analysis was performed using SAS software (version 9.4, SAS, Inc.). The chi-square test was used for clinical variables. Sensitivity, specificity, positive predictive value (PPV), and negative predictive value (NPV), along with their respective 95% confidence intervals (CIs), were calculated by comparing the results of ROP classification using indirect binocular ophthalmoscopy with those obtained from analysis of images captured by the portable camera, performed by a specialist. These metrics were calculated separately for images acquired with and without scleral indentation. The agreement between image exams and IBO results was measured by Cohen’s kappa coefficient κ. A value of 0 implied no agreement beyond chance, whereas a value of 1 corresponded to perfect agreement between IBO and the retinal images [20]. A significance level of 5% (p-value) was considered for all tests.

## Results

### Study population

**Fig. 2:**
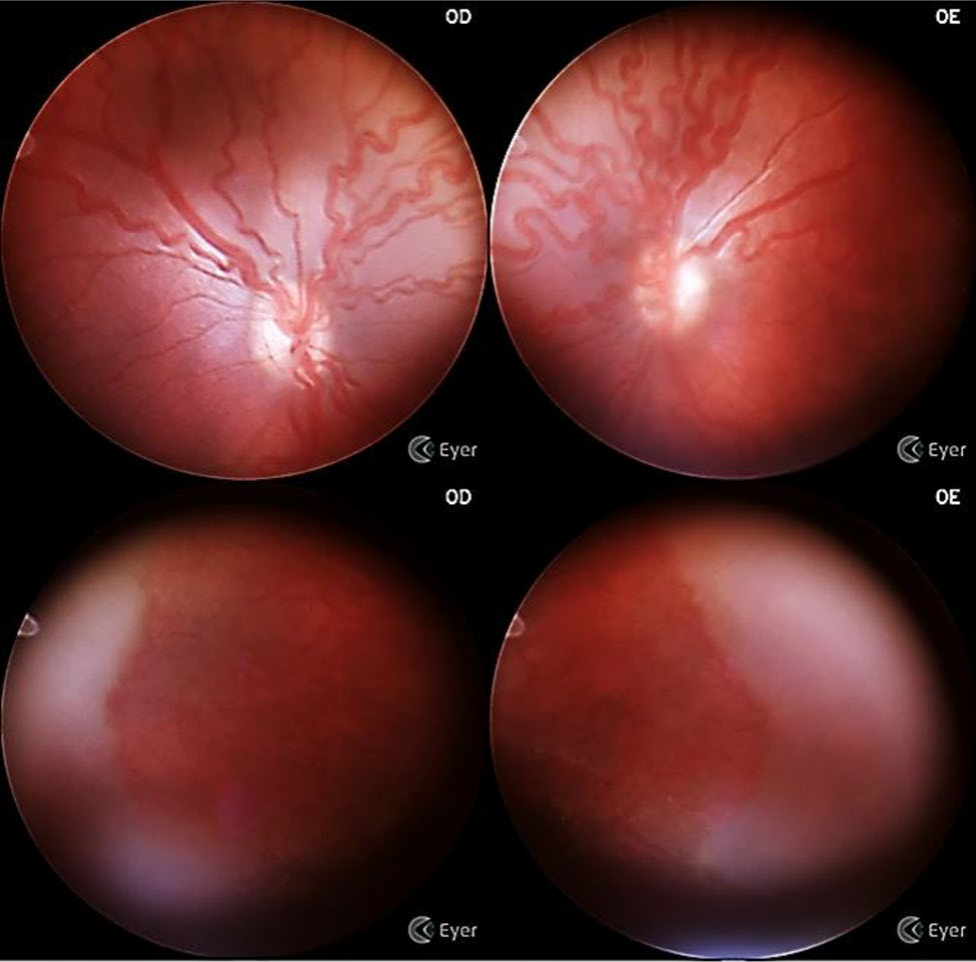
Example of image taken by Smartphone-based fundus imaging. Case of aggressive ROP (A-ROP) in Zone II

A total of 71 preterm infants (142 eyes) were included in the study. The mean GA was 29 ± 2.31 weeks, and the mean BW was 1238 ± 351 g. 52% of participants were female and 48% were male. Among the preterm infants, 14 (19.7%) developed ROP, with eight cases classified as ROP type 2 and six as type 1, requiring treatment (Table 1; Fig. 2).

**Table 1:** Demographic and clinical data of the study population

	Value
Number of neonates (eyes)	71 (142)
Mean GA (weeks), range	29 ± 2.31(25–36)
Mean BW (grams), range	1238 ± 351(580–2230)
Gender (Female), n (%)	37 (52)
No ROP (neonates), n (%)	57 (80.3)
ROP (neonates), n (%)	14 (19.7)
No ROP (eyes)	115
ROP (eyes)	27
ROP stage (eyes), n (%)	
Stage 1 Stage2 Stage 3 Stage 4 Stage 5 A-ROP	2 (7.4) 16 (59.2) 5 (18.5) 0 0 4 (14.8)
ROP zones (eyes), n(%)	
Zone 1 Zone 2 Zone3	2 (7.4) 17 (62.9) 8 (29.6)
Plus disease (eyes), n (%)	
Pre-plus Plus No plus	2 (1.4) 4 (2.8) 136 (95.8)
Non-threshold ROP	8 (16 eyes)
Threshold ROP	6 (11 eyes)

ROP: retinopathy of prematurity; BW: birth weight; GA: gestational age. A-ROP: aggressive ROP

Of the 142 eyes examined, 115 were classified as having no ROP, 2 (7.4%) as stage 1 ROP, 16 (59.2%) as stage 2, and 5 (18.5%) as stage 3. No eyes were classified as stages 4 or 5 ROP. For zone determination, two eyes (7.4%) were classified as Zone I, 17 (62.9%) as Zone II, and 8 (29.6%) as Zone III. Pre-plus disease was noted in 2 eyes (1.4%) and plus disease in 4 eyes (2.8%).

### Complications during examinations

The examination was well tolerated, with only one neonate (1.4%) presenting with bradycardia, characterized by a heart rate of approximately 80 bpm, during the image capture with scleral indentation. Immediately after the interruption of the maneuver, the physiological heart rate was reestablished. Even with improvement in the condition, it was decided to interrupt and reschedule the examination.

### Image analysis and diagnostic accuracy of the Smartphone-Based modified camera

**Fig. 3:**
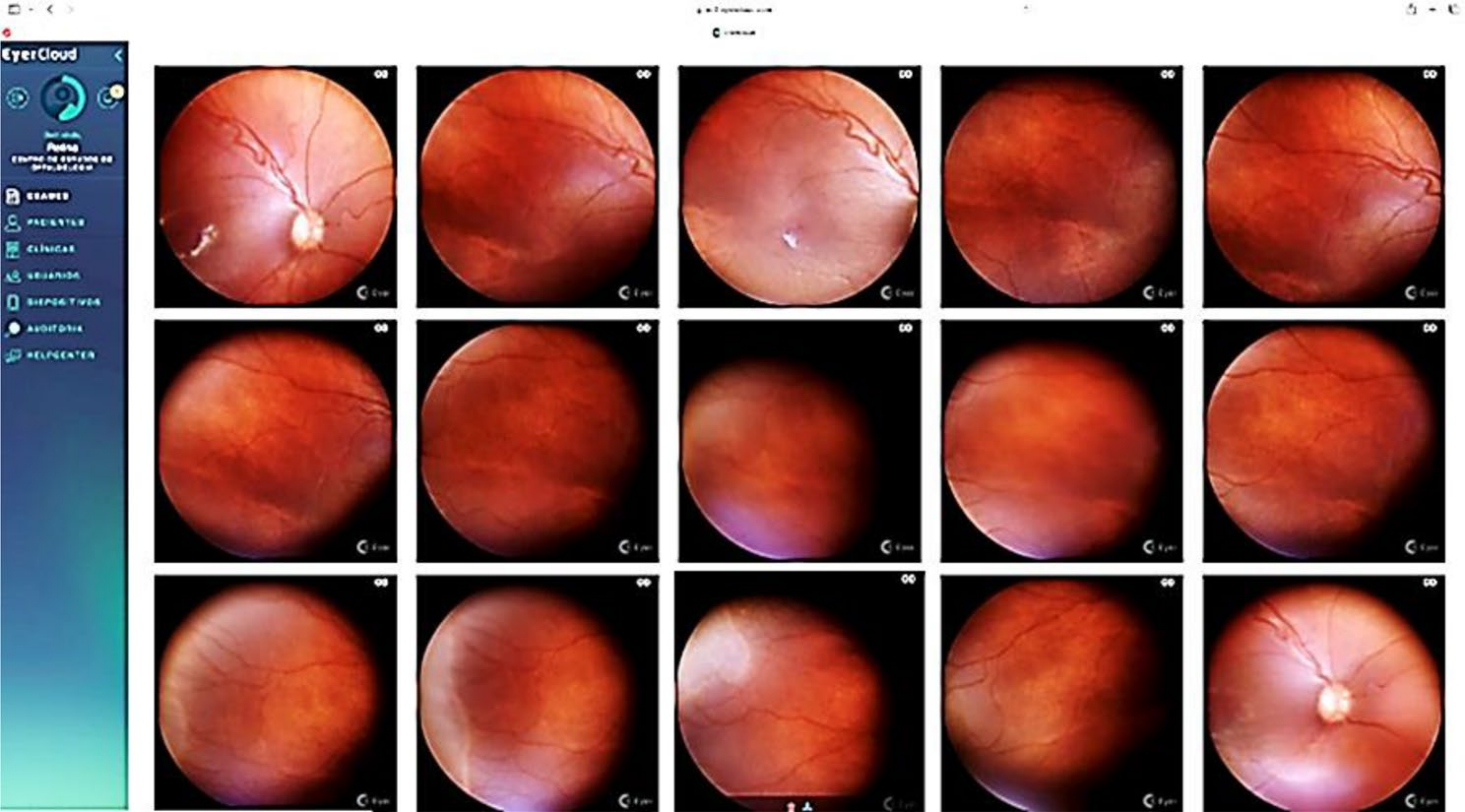
Eyercloud^®^ platform. Source: https://ec2.eyercloud.com.

The analysis of the image quality, performed by the ROP specialist, took into account the focus and the presence of artifacts that could hinder the identification of ROP signs. Of the 337 images evaluated, 233 (69%) were considered excellent, 104 (31%) were acceptable, and none were not gradable. The platform used to make the images available was Eyercloud (Fig. 3).

**Fig. 4:**
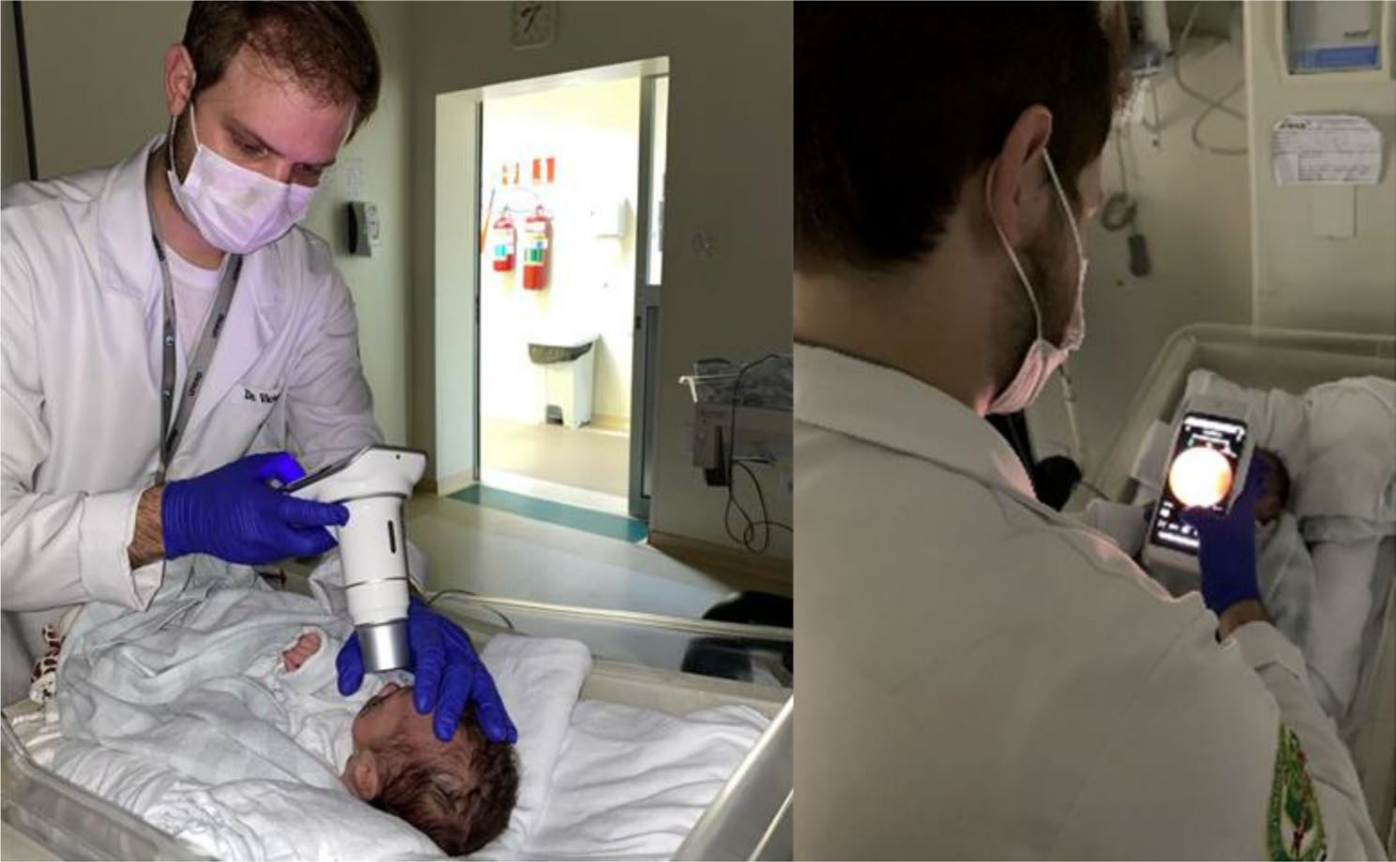
Capture of images with Smartphone-based portable camera without scleral indentation

The diagnostic performance of the Phelcom Eyer^®^ camera was evaluated against indirect binocular ophthalmoscopy (IBO). Without scleral indentation, the device demonstrated a sensitivity of 82% (CI 76.4–82.7), a specificity of 100%, a positive predictive value (PPV) of 100%, and a negative predictive value (NPV) of 96.7%. With scleral indentation, sensitivity increased to 91.3% (CI 86.7–91.4), and the NPV increased to 98.3% (Table 2; Fig. 4). The Kappa test, used to measure the level of agreement between the analyses, was 0.94%, considered almost perfect, according to the Landis & Koch criterion [20]. 

**Table 2:** Agreement between device and reference standard. Sensitivity, specificity, positive and negative predictive value, and kappa values ​​of the eyer portable camera, with and without using scleral indentation to capture images

Without scleral indentation	
Sensitivity (%)	82 (CI 76.4–82.7)
Specificity (%)	100
PPV (%)	100
NPV (%)	96.7
Kappa	0.94 (CI 0.78–0.99)
With scleral indentation	
Sensitivity (%)	91.3 (CI 86.7–91.4)
Specificity (%)	100
PPV (%)	100
NPV (%)	98.3
Kappa	0.94 (CI 0.87–1.00)

PPV: positive predictive value; NPV: negative predictive value; CI: 95% confidence interval

## Discussion

Indirect ophthalmoscopy performed by a trained specialist remains the gold standard for detecting retinopathy of prematurity [21]. However, screening performed using portable retinal cameras may establish itself as a viable and low-cost alternative, mostly in developing countries [22–24]. The present study is the first in Brazil to utilize a portable camera attached to a smartphone for identifying ROP, both with and without the use of scleral indentation.

The results demonstrate that the modified smartphone-based retinal camera (Phelcom Eyer) is a reliable tool for ROP screening, presenting high sensitivity and specificity when compared to indirect ophthalmoscopy. The use of scleral indentation, when necessary, enhanced sensitivity from 82 to 91.3% without compromising specificity, emphasizing its usefulness for visualizing the peripheral retina (zone III) as well. It is worth highlighting that, even without scleral indentation, no cases of ROP (type 1) that required treatment were missed since all cases occurred in zones I and II. Previous studies have validated digital imaging as a potential alternative method for ROP screening and documentation using various types of image capture devices [25–27]. However, the authors reported problems in identifying the stages and zones of ROP, especially in cases in zone III, which did not happen in the present study.

Smartphone-based retinal imaging systems offer a cost-effective alternative for public health, particularly in Brazil and other middle-income countries [22, 28]. In the Brazilian scenario, the two studies on ROP screening published to date have utilized high-cost portable systems, such as the Pictor Plus (Lanzelotte et al. ) [29] and the Clarity portable RetCam (Neves et al.) [10]. The disparity in costs is remarkable: for the price of a single portable RetCam, approximately 14 devices coupled to smartphones could be implemented in the Unified Health System (SUS) for screening via telemedicine, substantially expanding the country’s coverage of the exam.

Although an ophthalmologist performed smartphone imaging in this study, recent studies have shown that trained non-physician professionals can capture good-quality images and that telemedicine can be a viable alternative for ROP screening, especially in regions lacking specialists [10, 22, 23, 26, 30]. 

Another critical issue when evaluating screening tools is how easily the examiner can use them. In this study, all images were obtained by a single examiner with no previous camera experience to standardize the data and assess the learning curve. Initially, the acquisition of images of both eyes without scleral indentation took an average of 24.7 min. Although the IBO examination may be faster, after a few weeks of training, the time to acquire images of both eyes with the Phelcom Eyer^®^ camera was reduced to just 10 min. Furthermore, the camera’s intermittent illumination reduced newborn discomfort, which did not occur during examinations with the IBO’s intense, continuous light.

In addition, the ergonomic design of the Phelcon Eyer camera facilitated the acquisition of images in premature, extremely low-birth-weight neonates undergoing mechanical ventilation and incubation. In some newborns, the camera adaptation allowed for examinations inside the incubator, and in others, images could be captured without the use of a speculum. The presence of the continuous positive airway pressure (CPAP) device made it difficult to scan the temporal periphery because it hindered the camera’s inclination towards the nasal sector, a necessary maneuver for obtaining good zone II images without using scleral indentation. However, it did not prevent the detection of ROP but only increased the examination time.

Another benefit of using the Phelcon Eyer camera in this study was access to the EyerCloud platform, which made it possible to send the images captured via telemedicine for analysis by a specialist, and which will allow them to be used in the future to train artificial intelligence algorithms (such as PhelcomNet) for ROP detection. This functionality also enables the improvement of teaching for newcomers and specialists, as well as enhancing the engagement of parents of neonates during the monitoring of the disease’s progression.

This study also assessed potential complications that may arise during the examination. Without scleral indentation, the neonates had no complications such as apnea, bradycardia, or clinical instability. The infrared-guided positioning of the Eyer^®^ portable camera, which emits a flash of white light only during image capture, may have contributed to this outcome. Indirect ophthalmoscopy emits continuous, intense white light, which can cause discomfort and agitation in the neonate. A single neonate presented with bradycardia during scleral indentation, which was reversed after the maneuver was suspended.

Despite the promising results, this study had limitations. There was a single tertiary health center study with a small sample size. Multicenter studies could increase confidence in the results obtained. Furthermore, the field of view of smartphone-based systems is significantly smaller than that of IBO and wide-field retinal imaging cameras, which may make it difficult to capture peripheral images without scleral indentation, although this did not affect diagnostic accuracy in our study.

The strengths of this study included its prospective nature, the use of the same examiner, the non-use of scleral indentation except in cases of zone III ROP, and the application of the Kappa association measure to determine the degree of agreement, reliability, and accuracy in classifying the images.

Although Smartphone-Based Retinal Cameras are currently most widely used for ROP documentation, and the gold standard for ROP screening remains OBI performed by a trained specialist, with the development of appropriate protocols, improvement of devices, and training of examiners, they could become a low-cost teleophthalmology alternative, especially in underserved regions.

In conclusion, the Phelcon Eyer camera demonstrated excellent feasibility and accuracy in the diagnosis of ROP, particularly when combined with scleral indentation. Particularly in settings with limited resources and difficult access to specialists, its accessibility, portability, compatibility with telemedicine platforms, and ability to integrate with artificial intelligence systems make it an excellent strategy for improving the early detection of ROP and expanding screening coverage.

## Data Availability

No datasets were generated or analysed during the current study.
